# Lipocalin 2 over-expression facilitates progress of castration-resistant prostate cancer via improving androgen receptor transcriptional activity

**DOI:** 10.18632/oncotarget.11790

**Published:** 2016-09-01

**Authors:** Guanxiong Ding, Jianqing Wang, Chenchen Feng, Haowen Jiang, Jianfeng Xu, Qiang Ding

**Affiliations:** ^1^ Department of Urology, Huashan Hospital, Fudan University, Shanghai, China; ^2^ Department of Urology, Wake Forest University School of Medicine, Winston-Salem, North Carolina, USA

**Keywords:** castration-resistant prostate cancer, lipocalin 2, androgen receptor, cell proliferation, transcriptional activity

## Abstract

**Background:**

Castration-resistant prostate cancer (CRPC) is the lethal phenotype of prostate cancer. Lipocalin 2 (LCN2) is aberrantly expressed in many cancers including primary prostate cancer (PCa), but its role in CRPC has not been reported.

**Results:**

LCN2 expression was upregulated in human primary PCa and CRPC tissues. Overexpression of LCN2 promoted C4-2B and 22RV1 cell proliferation while knockdown of LCN2 markedly inhibited C4-2B and 22RV1 cell growth. LCN2 overexpression led to increased AR downstream gene SLC45A3 without upregulating AR expression. In the xenograft model, overexpression of LCN2 significantly promoted tumor growth.

**Methods:**

LCN2 expression was detected in primary PCa and CRPC tissues and cell lines C4-2B and 22RV1 using immunohistochemistry and western blotting, respectively. Serum LCN2 level was detected vi ELISA. Lentiviruses-mediated over-expression of LCN2 and LCN2 knockdown were performed in CRPC cell lines. Expressions of androgen receptor (AR) downstream genes was examined in cell lines, in CRPC tissues, and in animal models.

**Conclusion:**

LCN2 could facilitate cell proliferation of CRPC via AR transcriptional activity. LCN2 could be a novel target in CRPC.

## INTRODUCTION

Among all genitourinary cancers, lethal or high risk PCa is still a major cause of cancer-related death. The indolent phenotype of PCa, however, is usually indolent and can be perfectly curbed via surgery or radiotherapy. Lethal PCa, in most case at its castration-resistant stage, confers a poor prognosis and definitive therapy with durable effect is thus lacking. Several decades ago, when prostate cancer was introduced as an androgen-dependent disease, the treatment has been changed considerably [1]. Androgen deprivation therapy (ADT), as a standard care currently, is typically used through surgical castration or LHRH agonists/antagonists, when radiotherapy or surgery is failed. Despite the initial benefit upon the ADT, CRPC, which is known as hormone-refractory prostate cancer (HRPC) in the past, may be evolved from primary PCa 12-48 months following treatment [2, 3]. Regardless of whether testosterone reached castration level, the definition of CRPC is based on disease progression. In other words, CRPC can be defined by a constant increase in prostate-specific antigen (PSA) levels of serum, the development of preexisting disease, and/or the arriving of new metastases.

Multiple physiological and biochemical factors have been demonstrated in CRPC development. Mechanisms in physiology include extraprostatic synthesis of androgen in the suprarenal gland and/or local adipose tissue and intra-tumoral synthesis of androgen caused by increasing intracellular androgen levels of PCa cells [4–8]. Mechanisms in biochemistry include AKT phosphorylation and signaling as well as the pathway of vascular endothelial growth factor (VEGF) in the CRPC development [9, 10]. Even though it is suggested that multiple mechanisms result in the CRPC development, one common point lies in the AR-driven mechanism kept by the disease and the organ itself. For example, the fluid volume of the seminal plasma in men's ejaculation is produced mostly by the prostate and the differential, metabolic, proliferating and surviving processes of the epithelia and stroma of the prostate all result from the androgen binding to the AR [11].

Therefore, in order to tease out new strategies for curing patients with CRPC, deeper understanding is needed concerning the mechanisms of the molecule in CRPC. In an oncogene search in the transformation of SV40 from G0-arrested cell cultures of mouse kidney, LCN2, was discovered and featured for the first time, which was named as Neutrophil gelatinase-associated lipocalin (NGAL) [12]. As a superfamily member of the lipocalin, it is a 25 kDa protein containing the signal peptide that enables it to be secreted and form complexes with matrix metalloproteinase-9 (MMP-9) through disulfide bonds [13]. Elevated LCN2 has also been observed in a wide spectrum of solid tumor cells, including breast, ovarian and bladder [14–16]. We have previously demonstrated that LCN2 plays a role in facilitating cell migration and invasion in prostate cancer through inducing EMT by the axis of ERK/SLUG [17]. Thus, our study presently aims at how LCN2 is implicated in CRPC, proposing the potential of LCN2 in the therapy for CRPC.

## RESULTS

### LCN2 expression was upregulated in human PCa tissues

**Figure 1:**
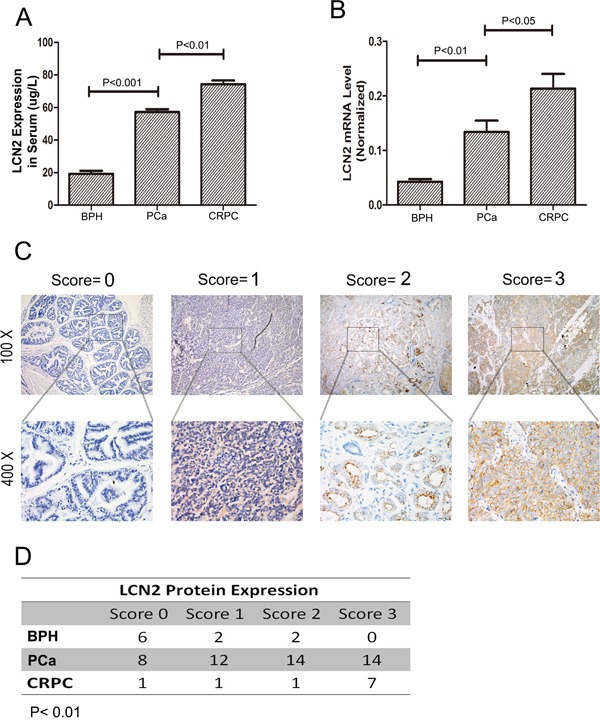
LCN2 expression was upregulated in human PCa tissues **A.** The LCN2 levels were significantly upregulated in serum samples from CRPC patients than in those from PCa patients and BPHs. **B.** LCN2 mRNA levels were upregulated in tissue samples from CRPC patients than in those from PCa patients and BPHs. **C.** The LNC2 protein showed a typical cytoplasma staining pattern in prostate tissues via IHC method(original magnification, ×100 or ×400). Each case was given a scale of 0 to 3 (0=no staining, 1=weak staining, 2=intermediate staining, and 3=strong staining). **D.** Enhanced immunoreactivity of LCN2 in CRPC tissues was observed in CRPC tissues.

The expression level of LCN2 was examined using qRT-PCR in a cohort of 68 individuals, including 48 PCas, 10 CRPCs and 10 BPHs. As we have previously shown that PCa expressed higher LCN2, we have in the current study investigated whether LCN2 was further upregulated in CRPC. Also, as a secretory protein, serum LCN2 levels from different groups were analyzed by ELISA method. We found that serum LCN2 levels were significantly upregulated in CRPC patients than in PCa or BPH patients (Figure 1A). Consistent with the ELISA results, the increased expression of LCN2 in CRPC tissues was shown (Figure 1B). To further confirm this result, we measured the LCN2 expression using Immunohistochemical staining (IHC). The LCN2 protein showed a typical cytoplasm staining pattern in prostate tissue (Figure 1C). More importantly, substantial enhanced immunoreactivity of LCN2 in CRPC tissues was observed among three groups (Figure 1D).

### High expression of LCN2 promoted proliferation of CRPC cells

**Figure 2:**
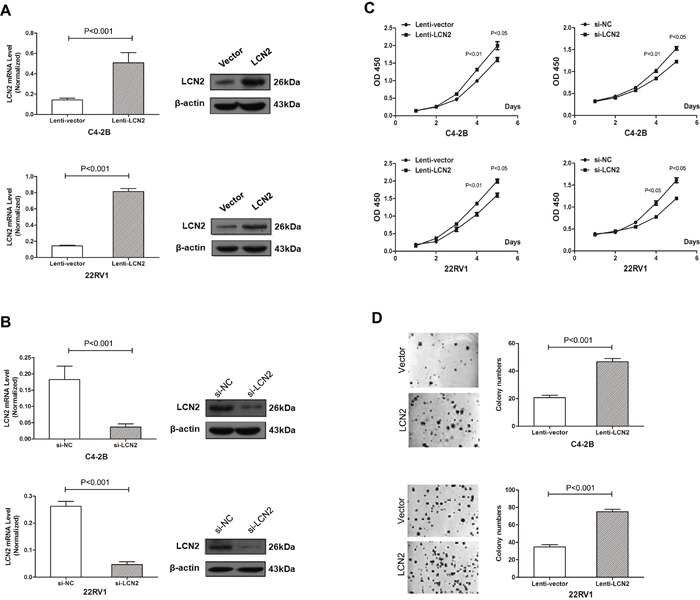
High expression of LCN2 promoted CRPC cell proliferation **A.** Expression of LCN2 was dramatically increased in C4-2B and 22RV1 cells infected with a lentivirus encoding the LCN2 gene. **B.** Expression of LCN2 was decreased in C4-2B and 22RV1 cells afected with the LCN2 siRNA. **C.** A cell proliferation assay revealed that overexpression of LCN2 significantly enhanced the growth rate of C4-2B and 22RV1 cells. The knockdown of LCN2 markedly inhibited cell growth in C4-2B and 22RV1 cells. **D.** A colony formation assay confirmed that LCN2 overexpression promotes the proliferation of C4-2B and 22RV1 cells.

Over-expression of LCN2 in two CRPC cell lines (C4-2B and 22RV1) was introduced to clarify the role of LCN2 in cell growth. Expression of LCN2 was dramatically increased in C4-2B and 22RV1 cells infected with a lentivirus encoding the LCN2 gene (Figure 2A). Cell proliferation assay revealed that overexpression of LCN2 significantly enhanced the growth rate of C4-2B and 22RV1 cells (Figure 2C). Colony formation assay confirmed that LCN2 overexpression promoted proliferation of C4-2B and 22RV1 cells (Figure 2D). Furthermore, the knockdown of LCN2 was performed by siRNAs, which markedly inhibited cell growth in C4-2B and 22RV1 cells (Figure 2B, 2C). Collectively, these results suggested that LCN2 promoteed proliferation in CRPC cells.

### SLC45A3 expression was correlated with LCN2

**Figure 3:**
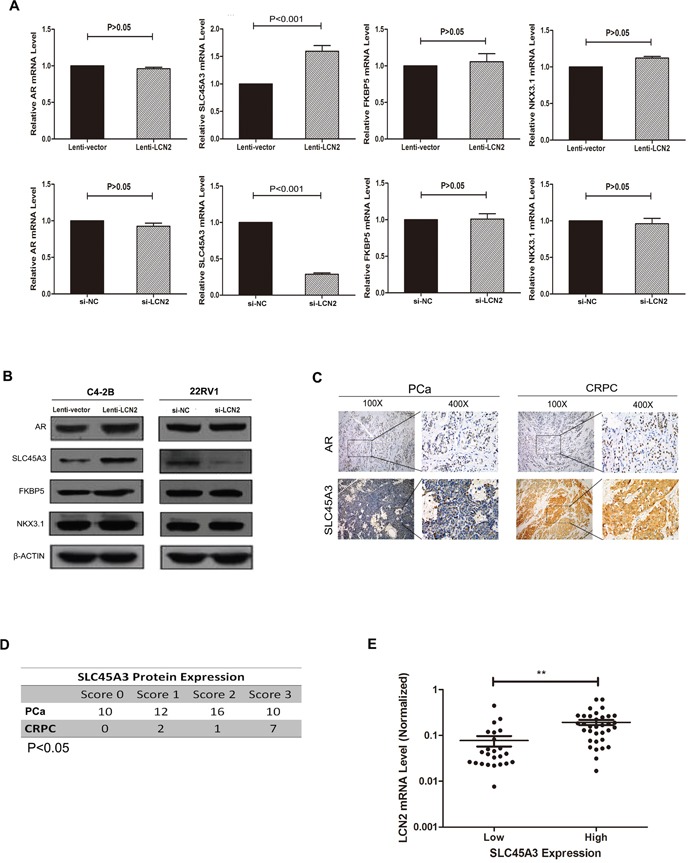
SLC45A3 expression was correlated with LCN2 **A, B.** SLC45A3 expression was increased in LCN2 over-expressed C4-2B cells and was decreased in LCN2 knockdown 22RV1 cells. LCN2 mRNA and protein levels were determined with RT-qPCR and western blot, respectively. **C.** The AR protein showed a typical nucleus staining pattern in prostate tissues via IHC method(original magnification, ×100 or ×400). **D.** The SLC45A3 protein were upregulated in CPRC tissues compared with PCas (P<0.05). **E.** SLC45A3 protein expression was positively correlated with LNC2 mRNA levels.

Growing evidence has indicated that castration resistance was partially due to the enhancement of AR transcriptional activity. To investigate the mechanism of LCN2 upregulation in CRPC, we detected the expression of AR downstream genes in cell lines and tissues. We examined SLC45A3, FKBP5, NKX3.1, as well as AR expressions in LCN2-overexpression and -knockdown cells. SLC45A3 expression was dramatically increased when LCN2 was over-expressed in C4-2B cells. Consistently, the knockdown of LCN2 in 22RV1 cells significantly reduced the expression of SLC45A3. However, this was not the case for AR, FKBP5 and NKX3.1 (Figure 3A and 3B). To further demonstrated this relationship among LCN2, AR and SLC45A3, protein expression was measured in CRPC, PCa and BPH. Accordingly, SLC45A3 expression were improved in CRPC tissues and SLC45A3 expression was positively correlated with LNC2 mRNA levels (Figure 3C, 3D and 3E). These data suggested that SLC45A3 was involved in the LCN2-induced castration resistance in CRPC.

### LNC2 promoted CRPC growth and enhanced SLC45A3 expression *in vivo*

**Figure 4:**
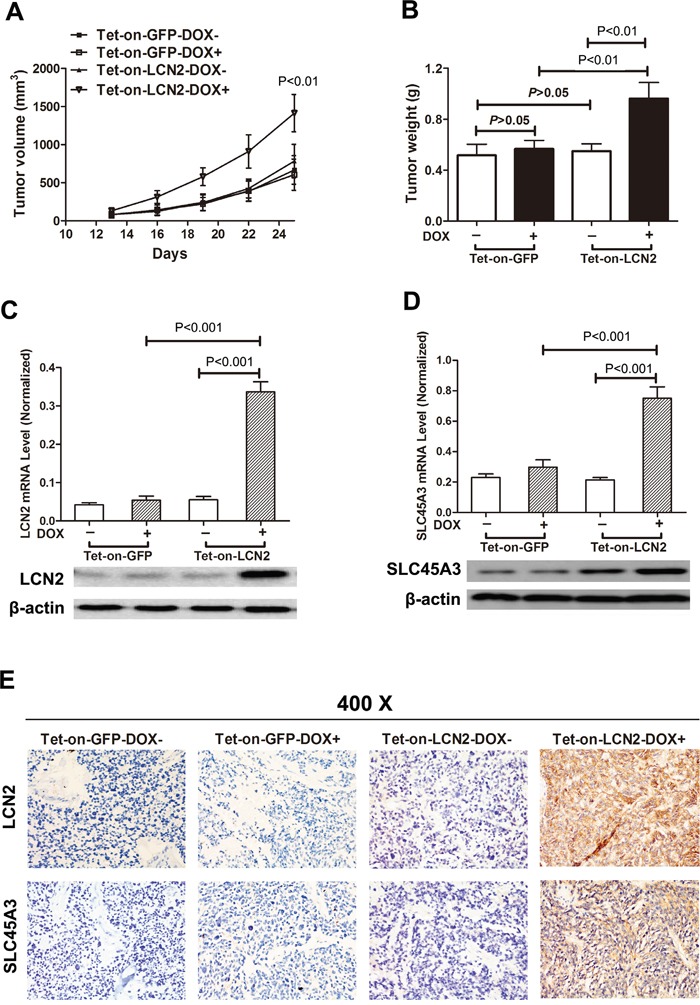
LNC2 promoted CRPC cells growth and enhanced SLC45A3 expression *in vivo* **A, B.** LCN2 overexpressed 22RV1 cells were injected subcutaneously at right flanks of each mouse and LCN2 expression was induced with DOX in drinking water via TET-ON system. Compared with the control groups, overexpression of LCN2 promotes tumor growth with remarkable tumor volume and weight in nude mice. **C.** LCN2 expression was determined by RT-qPCR and western blot and LCN2 was only expressed in LCN2 tet-on group. **D.** SLC45A3 expression was determined by RT-qPCR and western blot and SLC45A3 was only expressed in LCN2 tet-on group. **E.** LCN2 and SLC45A3 expression were determined by IHC and were only expressed in LCN2 tet-on group.

To further determine the biological significance of the above results, tumor formation assays were conducted *in vivo*. LCN2-overexpressed 22RV1 cells were injected subcutaneously at right flanks of each mouse and LCN2 expression was induced with DOX in drinking water via TET-ON system. Compared with control groups, overexpression of LCN2 promoted tumor growth with remarkably increased tumor volume and weight in nude mice (Figure 4A and 4B). LCN2 overexpression was validated in xenograft tumors and LCN2 induced SLC45A3 upregulation was demonstrated (Figure 4C, 4D and 4E).

## DISCUSSION

As a major problem concerning men's health, CRPC plays an important role in the compromised prognosis of the tumor due to its aggressiveness. As a result, the identification and the characterization of CRPC and its significant regulators are extremely necessary. We have previously reported that LCN2 is closely related to the invasiveness of cells of prostate cancer [17]. Based on present results, it can be confirmed that levels of LCN2 is substantially up-regulated in samples of serum gained from patients with CRPC compared with those from PCa patients and the normal controls and enhanced immunoreactivity of LCN2 in CRPC tissues was observed in CRPC tissues. Other studies report that LCN2 is overexpressed in the intestine in colitis patients and acts as a negative prognostic indicator in colorectal cancer [18]. Furthermore, several studies have also shown that LCN2 is present in the urine of patients with breast cancer and in tissue homogenates from gastric cancer patients [19, 20]. Besides, we have also found that overexpression of LCN2 significantly enhances the growth rate of CRPC cell lines. According to previous reports, the influence of LCN2 on cell proliferating and differentiating processes can be confirmed together with its involvement in cancer development [21]. Earlier studies have also demonstrated that LCN2 overexpression is associated with proliferation of human ovarian cancer cell lines [22].

Based on the hypothesis, it is suggested that mechanisms like variations of genes, epigenetic alteration, and transcriptional and translational regulation changes may result in the proliferation of the tumor without the binding of ligand. The AR gene is a steroid hormone receptor found in both benign and malignant prostate epithelial cells. AR is a transcription factor, which is activated by the ligand with three distinct domains: the domain of DNA-binding (DBD); the domain of C-terminal ligand-binding (LBD); and the domain of N-terminal transactivation (NTD), which drive transcriptional activity [23, 24]. To investigate the mechanism of LCN2 upregulation in CRPC, the expression of AR downstream genes in cellular lines and tissues are detected. It is reported that castration resistance is due to continuous upregulated AR expression or AR transcriptional activity. In our results, LCN2 overexpression leads to high expression of AR downstream gene SLC45A3 without AR upregulation. That result suggested high AR transcriptional activity devote to LCN2 mediated CRPC progression.

Based on preclinical experiments as well as targeting therapeutics of AR, it has been widely accepted that there is no independence of CRPC in terms of the transcriptional signaling of AR [25, 26]. When ADT becomes less successful, certain different mechanisms can be proposed in terms of patients' response of developing resistance. Specifically speaking, these mechanisms include AR overexpression [27] or coactivator overexpression [28] that sensitizes AR to reduced androgen level physiologically, including point mutations that may lead to the promiscuous activation of AR by nonandrogenic steroids with the relative abundance; the AR being activated/sensitized through the protein phosphorylation [29]; and the androgen de novo production on its own of the tumor [6]. We have also found that overexpression of LCN2 promotes tumor growth in nude mice. LCN2 overexpression is validated in xenograft tumors and LCN2 induces AR transcriptional. Due to the crucial role of the transcriptional activation of AR in cancer development, an effective therapeutic target is essential to cure patients with CRPC.

In summary, LCN2 can be a biomarker for both disease progression of CRPC and indicator of AR transcriptional activity. Whether inhibition of LCN2 contributes to treatment of CRPC warrants further investigation.

## MATERIALS AND METHODS

### Study population

Individuals included in this study underwent surgery between 2012 and 2015 at Huashan Hospital, Fudan University. A total of 48 primary PCa patients (sensitive to ADT), 10 CRPC patients and 10 benign prostatic hyperplasia patients (BPH) were prepared for the analysis of LCN2 expression in both tissue and blood samples. BPH samples were derived from patients with dysuria by TURP (transurethral resection of the prostate) and PCa samples were derived from patients with a diagnosis of PCa by radical prostatectomy(RP) or TURP. CRPC samples were derived from CRPC patients with urinary retention by palliative TURP. The tissues were collected according to the anatomical site of biopsy and a subsequent pathological confirmation was performed. These tissue samples were immediately snap-frozen in liquid nitrogen. Blood samples of the patients were collected before operation and stored at −80°C. The Clinical Research Ethics Committee of Fudan University Cancer Hospital approved the research protocols and written informed consents were obtained from the participants.

### Cell culture

Human CRPC cell lines, C4-2B and 22RV1, were commercially available and were purchased from American Type Culture Collection (ATCC). Cells were cultured in RPMI 1640 media with 10% fetal bovine serum (FBS). HEK-293T cell line were purchased from ATCC and were cultured in DMEM (Gibco, NY) and 10% FBS. Cells were incubated with 5% CO_2_ at 37°C.

### RNA extraction and quantitative real-time RT-PCR

Total RNA was extracted using the TRIzol reagent (Invitrogen, USA) from 50 mg of tissue samples. The specific steps according to the manufacturer's instructions. The concentrations were determined using a NanoDrop ND-1000 (NanoDrop, USA). cDNA was synthesized with the PrimeScript RT reagent kit (TaKaRa, Japan) using 500ng total RNA as template. qPCR analyses were conducted to quantitate mRNA relative expression using SYBR Premix Ex Taq (TaKaRa, Japan) with beta-actin as an internal control. PCR was performed using an ABI 7900HT instrument (Applied Biosystems, USA). The primers for RT-qPCR were shown in the Supplementary Table S1.

### Vector constructs and lentivirus transduction

The open reading frame (ORF) of LCN2 was amplified by nested PCR and cloned into the pLVX-IRES-Neo vector (Clontech, USA). The primers and endonuclease sites used for the vector constructs are shown in the Supplementary Table S2. Virus particles were harvested 48 hours after cotransfecting pLVX- LCN2-1 with the packaging plasmid ps-PAX2 and the envelope plasmid pMD2G into HEK-293T cells using Lipofectamine 2000 reagent (Invitrogen, USA). C4-2B and 22RV1 cells were infected with recombinant lentivirus-transducing units plus 6 μg/mL polybrene (Sigma, USA). The siRNA target sequences (5′-3′) were as follows: human LCN2, GGAGCTGACTTCGGAACTAAA.

### Cell proliferation assay and colony formation assay

Cell proliferation was quantified using the Cell Counting Kit-8 (CCK-8; Dojindo Laboratories, Japan) according to the manufacturer's instructions. For the colony formation assays, 1,000-2000 cells per well of C4-2B and 22RV1 were incubated in medium containing 10% FBS for 2 weeks. The colonies were fixed with methanol and stained with 0.1% crystal violet in 20% methanol for 20 minutes. The number of colonies containing more than 30 cells was counted using an inverted microscope.

### Xenograft and intravenous tumor model

Male BALB/c athymic nude mice at 5 weeks of age (Vital River, China) were bred in licensed SPF (special pathogen-free) grade laboratory. A total of 2 ×106 cells (C4-2B and 22RV1, stably expressing LCN2 or the vector control) in 200μl of PBS were injected subcutaneously at right flanks of each mouse. After transplantation, the growth of the subcutaneous tumors was assessed twice a week. Tumor size was monitored by measuring the length and width with callipers, and volumes were calculated with the formula: (L×W2) × 0.5, where L is length and W is width of each tumor. The mice were sacrificed after a period of 5-7 weeks, and the weight of subcutaneous tumors were recorded. All protocols were approved by Fudan University animal ethics committee.

### Elisa assay

The plasma samples which had been stored in freezer of −80°C were thawed on ice and centrifuged at 10000g for 5 minutes at 4°C to remove any debris. Repeated freeze thaw cycles were avoided. Full length level of LCN2 in circulation was measured using the Human Lipocalin-2/NGAL Quantikine ELISA Kit (R&D Systems, USA).

### Western blot

Harvested proteins were first separated by 10% sodium dodecyl sulphate-polyacrylamide gel electrophoresis and then transferred to nitrocellulose membranes (Bio-Rad Laboratories, USA). The membranes were blocked with 5% nonfat milk and incubated with antibodies. The membranes were subsequently incubated with a goat anti-mouse horseradish peroxidase secondary antibody (Sigma, USA). The protein complex was detected using enhanced chemiluminescence reagents (Pierce, France). Endogenous beta-actin was used as the internal control. LCN2 antibody was purchased from R&D Systems, USA. AR, PSA, SLC45A3, FKBP5 and NKX3.1 were purchased from Santa Cruze, USA.

### Immunohistochemical staining

Immunohistochemical staining was performed on 4μm sections of paraffin-embedded tissues. The slides were incubated in LCN2 antibody (R&D Systems, USA) diluted 1:100 at 4°C overnight and incubated in second antibody (DAKO, Demark) at 37°C for 30 minutes. Then the slides were stained with the avidin–biotin-peroxidase method with DAB (Diaminobenzidine) and counterstained with hematoxylin. The specific steps were performed using the EnVision™ FLEX High pH visualisation system according to the manufacturer's instructions (DAKO, Demark). Each case was given a scale of 0 to 3 (0=no staining, 1=weak staining, 2=intermediate staining, and 3=strong staining).

### Statistical analyses

The results are presented as the mean values ± SEM. Differences between groups were estimated using the χ^2^ test and Student's t test. Relationships were explored by Spearman's correlation. A value of P < 0.05 was considered statistically significant. SPSS 16.0 package (IBM, USA) and Graphpad prism 5.0 software (GraphPad Software, USA) were used for statistical analyses and scientific graphing, respectively.

## SUPPLEMENTARY TABLES


