# Supplementary material for: Lipocalin 2 over-expression facilitates progress of castration-resistant prostate cancer via improving androgen receptor transcriptional activity
Source: Oncotarget. 2016 Sep 1;7(39):64309–17. doi: 10.18632/oncotarget.11790 (PMC5325444; doi:10.18632/oncotarget.11790)
Supplement: Supplementary file 1 [file oncotarget-07-64309-s001.pdf]

Lipocalin 2 over-expression facilitates progress of castration-resistant prostate cancer via improving androgen receptor transcriptional activity

SUPPLEMENTARY TABLES

Supplementary Table S1: Primers for the RT-qPCR

Primer name	Sequence(5' to 3')
LCN2-F	CCACCTCAGACCTGATCCCA
LCN2-R	CCCCTGGAATTGGTTGTCCTG;
AR-F	GACGACCAGATGGCTGTCATT
AR-R	GGGCGAAGTAGAGCATCCT
SLC45A3-F	CCTTCACGCTGTTTTACACGG
SLC45A3-R	CGCCTTCATCATAGTGTCTCC
FKBP5-F	CTCCCTAAAATTCCCTCGAATGC
FKBP5-R	CCCTCTCCTTTCCGTTTGGTT
NKX3.1-F	CCCACACTCAGGTGATCGAG
NKX3.1-R	GAGCTGCTTTCGCTTAGTCTT

Supplementary Table S2: Primers for the cloning of LCN2 ORF

Primer name	Sequence(5' to 3')	Product size(bp)
LCN2-OUT-F	TGCTTCCTCGGCCCTGAAAT	749
LCN2-OUT-R	TTAGCAGACAAGGTGGGGCT	
LCN2-IN-F	CCTGAATTCATGCCCCTAGGTCTCCTGTG	615
LCN2-IN-R	CCTGGATCCTCAGCCGTCGATACACTGGTC	

* Sequences underlined are sites for restriction enzyme.
